# Chemical Characterization and Cytotoxic/Antibacterial Effects of Nine Iranian Propolis Extracts on Human Fibroblast Cells and Oral Bacteria

**DOI:** 10.1155/2022/6574997

**Published:** 2022-04-06

**Authors:** Mohsen Yazdanian, Mohammad Nima Motallaei, Elahe Tahmasebi, Hamid Tebyaniyan, Mostafa Alam, Kamyar Abbasi, Alexander Seifalian, Reza Ranjbar, Alireza Yazdanian, Hamideh Mahmoodzadeh Hosseini, Mehrdad Moosazadeh Moghaddam

**Affiliations:** ^1^Research Center for Prevention of Oral and Dental Diseases, Baqiyatallah University of Medical Sciences, Tehran, Iran; ^2^Islamic Azad University, Science and Research Branch, Tehran, Iran; ^3^Department of Oral and Maxillofacial Surgery, School of Dentistry, Shahid Beheshti University of Medical Sciences, Tehran, Iran; ^4^Department of Prosthodontics, School of Dentistry, Shahid Beheshti University of Medical Sciences, Tehran, Iran; ^5^Nanotechnology and Regenerative Medicine Commercialization Centre (NanoRegMed Ltd), The London Bioscience Innovation Centre, London, UK; ^6^School of Dentistry, Baqiyatallah University of Medical Sciences, Tehran, Iran; ^7^Department of Veterinary, Science and Research Branch, Islamic Azad University, Tehran, Iran; ^8^Applied Microbiology Research Center, Systems Biology and Poisonings Institute, Baqiyatallah University of Medical Sciences, Tehran, Iran; ^9^Applied Biotechnology Research Center, Baqiyatallah University of Medical Sciences, Tehran, Iran

## Abstract

Multimicrobial infections caused by pathobionts are called dysbiotic multimicrobial illnesses. Commercial mouthwashes, such as chlorhexidine, have negative side effects that can prevent tooth decay and infection. The present study aimed to determine the antifungal, antibacterial, and cytotoxicity characteristics of the propolis extracts from different areas (Iran). The ethanolic extract of propolis was prepared. GC/MS carried out the characterization to determine the thymol, carvacrol, and menthol extracts, and also, total phenol and flavonoid were assed for all samples. The antimicrobial and antibiofilm effects were evaluated against *S. mutans*, *S. mitis*, *S. salivarius*, *L. acidophilus*, *E. coli*, *S. aureus*, and *C. albicans.* The cytotoxic effect of extracts was measured on human fibroblast cells by MTT test. The MIC values in mg mL^−1^ were ranged as follows: *S. salivarius* (0.003 to 0.048), *S. mutans* (0.003 to 0.029), *S. mitis* (0.007 to 0.058), *L. acidophilus* (0.007 to 0.117), *C. albicans* (0.014 to 0.234), *E. coli* (0.007 to 0.058), and *S. aureus* (0.007 to 0.058), while MBC were, respectively, *S. mutans* (0.007 to 0.058), *S. salivarius* (0.007 to 0.117), *S. mitis* (0.007 to 0.117), *L. acidophilus* (0.014 to 0.234), *C. albicans* (0.029 to 0.468), *E. coli* (0.014 to 0.234), and *S. aureus* (0.007 to 0.117). Cariogenic bacteria and *Candida albicans* were demonstrated to be resistant to propolis extracts. Therefore, propolis extracts may make good mouthwashes.

## 1. Introduction

A variety of factors contribute to dental caries. Biological fermentation produces lactic acid, which contributes to dental caries. The presence of dental biofilm promotes the progression of periodontal disease and caries [1]. Public health issues such as dental caries affect millions [2]. It is believed that bacteria, primarily *Streptococcus mutans*, contribute to the initiation of caries. However, caries does not always require the presence of bacteria for its development [1, 3]. Tooth decay is dependent upon *Streptococcus mutans'* ability to produce extracellular polysaccharides (mainly glucans). The bacteria use glucosyltransferases to turn nutritious carbohydrates (GTF) into glucans [1]. *S. mutans* has been successfully removed from the oral cavity after repeated attempts. Dental cavities can often be reduced with antibiotics such as ampicillin, penicillin, and tetracycline. These compounds are also associated with negative effects, such as increased susceptibility to bacteria, diarrhea, vomiting, and tooth discoloration when ingested in large quantities. This plant has broad spectrum antibacterial activity against oral bacteria, including *Sanguinaria canadensis*. A unique oral product is due to its powerful antibacterial properties. The use of this drug was limited due to its association with oral leukoplakia. In light of these challenges, more research is needed on natural antibacterial materials that are safe and effective against oral microorganisms [4]. Propolis is a brownish waxy product produced by the honeybee from plant leaves, buds, and exudates. Propolis, known from ancient times, possesses anti-inflammatory, antimicrobial, antioxidant, hepatoprotective, immunostimulating, and cytostatic properties [5].

Pollen, flavonoids, phenolic acids, waxes, and aromatic balsam constituents of propolis are what it is primarily made up of. Depending on where and how it is made, propolis varies in composition based on what kind and what kind of plants are used for making it [5]. Flavonoids have an essential role in the biological activity of propolis [5]. The biochemical properties of flavonoids are binding biological polymers and heavy metal ions, scavenging free radicals and catalysis of electron transport [5]. The flavonoids inhibit the integration of uridine, thymidine, and leucine into tumoral cells and inhibit DNA synthesis and cause the antitumoral effect of propolis [5]. Bees use propolis to seal their hives and thus check the entry of microbes. The synergistic effect of its compounds causes antimicrobial properties of propolis [5]. Propolis works against harmful bacteria by affecting the integrity of the membrane and thus inhibiting bacterial enzyme activity and motility. Propolis is effective against antibiotics-resistant bacteria [5]. Propolis has a wide range of applications. It contains urinary tract infection, cancer, treatment of open wounds, influenza, sinus congestion, gastritis, ear disease, periodontal disease, intestinal infections, arthritis, headaches, Parkinson's disease, conjunctivitis, and warts [5]. Propolis is used against invasive fungi, bacteria, and even larvae [6]. Several studies have demonstrated the antimicrobial activities of propolis [6–21]. The effectiveness of propolis against *Streptococcus mutans* was reported by many studies [1]. In this study, it was examined whether propolis alcoholic extract from different parts of Iran has any effect on normal fibroblast cells and how effective it is at controlling oral microbes.

## 2. Materials and Methods

### 2.1. Materials

In this study, we were interested in testing how propolis extract affected different bacteria that cause oral infections. This led to the selection of a range of bacteria that cause oral disorders. Iranian University of Medical Sciences provided *S. salivarius*, *S. mutans*, *S. mitis*, *C. albicans*, *L. acidophilus*, *S. aureus*, *E. coli*, and human fibroblast cells. MTT Kit was obtained from Bioidea (Iran). YPD broth, BHI agar and broth, and crystal violet were provided from Merck (Darmstadt, Germany). Trypsin, DMEM, PBS, FBS antistreptomycin, and beta-glycerol were bought from Gibco (New York, USA). DMSO was obtained from Sigma-Aldrich.

### 2.2. Propolis Sampling and Extraction

#### 2.2.1. Propolis Sampling

**Figure 1:**
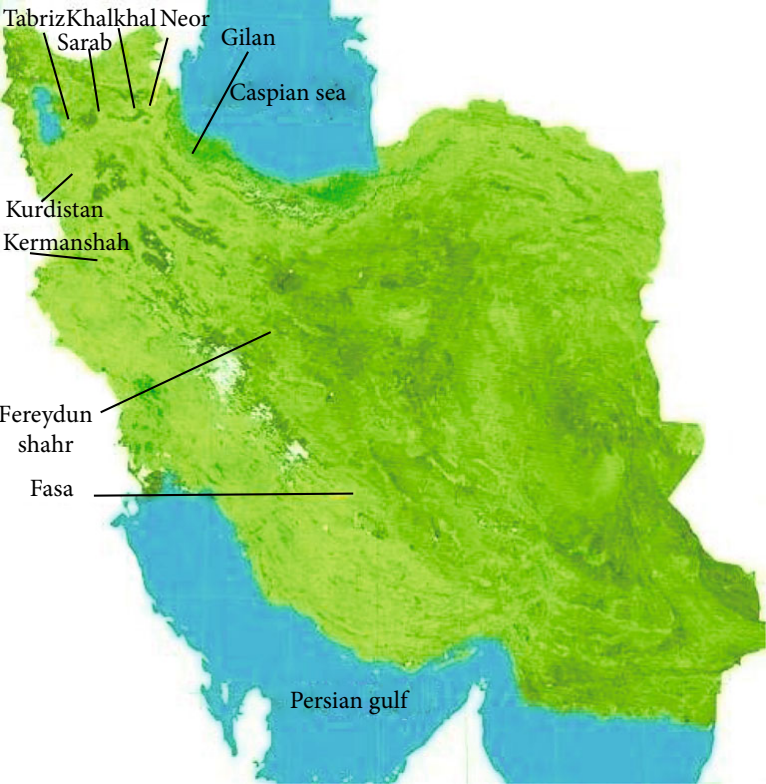
Nine samples of propolis were gathered from various parts of Iran.

Raw propolis was collected in 2020 from Tabriz (East Azerbaijan), Kurdistan, Khalkhal (Ardabil Province), Sarab (East Azerbaijan Province), Neor Lake (Ardabil Province), Fasa (Fars Province), Qaleh Rudkhan (Gilan Province), Fereydun Shahr (Isfahan Province), and Kermanshah (Figure 1).

#### 2.2.2. Propolis Extract Preparation

Samples were frozen (-20°C) and then grounded. Raw propolis samples were extracted (under stirring (by tenfold volume of ethanol (70%)) in firmly closed flasks in the dark environment, at ambient temperature for three days. Then the suspensions were frozen (-20°C, 24 h) and filtered to remove less soluble substances and waxes (Whatman filter paper (No. 1). This process was repeated (three times). What remains at the end is ethanol extract of propolis (EEP). By a rotary evaporator (rotary evaporator), the solutions were evaporated (under reduced pressure at 64°C) to near dryness. Then the solutions were freeze-dried to obtain a powder [3].

### 2.3. Gas Chromatography/Mass Spectrometry (GC/MS)

The GC/MS was performed using a GCMS (QP2010S (Shimadzu, Japan)). In 10 mL of 50% ethanol, freeze-dried propolis (1 g) was dissolved. In this experiment, EEP (25 mg) was evaporated under nitrogen conditions, then derivatized (by one percent TMCS, 100 L BSTFA, 50 g pyridine, and one cc hexane after one day), and dissolved in one cc hexane. As a carrier, helium gas (one liter) was used (at a flow rate of 0.05 mL/min) (in a splitting ratio of 1 : 25). Capillary column was connected to a quadrupole mass spectrometer. Specifically, the head pressure was adjusted at 53.1 kPa, the injector temperature was adjusted at 230°C, and the transfer line heater temperature was adjusted at 250°C. With GC/MS Postrum Analysis, the mass spectra were as follows: 1-s scan time, 35–450 m/z scan range, 220°C source temperature, 70 eV electron energy, and 3-min filament delay time [22].

### 2.4. Total Phenolic Compounds Analysis

This study was conducted using the Folin-Ciocalteu spectrophotometric technique with gallic acid as the standard. Extraction was carried out in ethanol (0.1 mg.min^−1^) with a concentration of 0.01. In the next step, sodium carbonate (7.5 percent) and Folin-Ciocalteu solution (2.5 mL) (10 percent) were added to the solution. A 50-degree bath was used to soak the solution for 5 minutes. Spectrophotometers (765 nm) were used to measure absorbances. In this study, gallic acid standard curves (mg EGA/g) were compared with the raw data. This process was repeated three times [23].

### 2.5. Flavonoid Content Analysis

A spectrophotometer (415 nm) was used to measure the flavonoid content of EEP. Methanol was mixed 1 : 1 with aluminum chloride 2.0 percent to create the solutions. Standard solutions of quercetin were used to set the curves. A blank sample was evaluated for flavonoid content (mg EQ/g) and represented as quercetin equivalents [23]. Samples were analyzed three times [23].

### 2.6. MTT Assay

Several doses of EEP (12.5 to 0.006 mg/mL) were used in 96-well plates to culture human gingival fibroblasts. An assay for the determination of cell survival was performed using 3-(4,5-dimethylthiaziazol-2-yl) 2,5-diphenyl tetrazolium bromide. The cells were plated at 2105 cells/mL in each well. In the following step, EEP samples in DMEM (without serum) (100 L/well) were diluted to a variety of concentrations. Cells without extracts served as a control. A humid environment containing 5% CO2 with 37°C and a humid atmosphere was used for 24 hours to incubate the colonies. During the next phase, cell growth was measured using MTT solution (5 mg/mL). A 5% CO_2_ atmosphere and 37°C were used to incubate plates with MTT solutions for four hours. Dimethylsulfoxide (DMSO) was added to the well's medium. Crystals were dissolved in DMSO. ELISA reader (EL X 808) was used to examine the plates after 10 minutes at room temperature (lambda wavelength 570 nm, reference wavelength 630 nm). An MTT-based technique was used to determine mitochondrial activity after 24 and 48 hours of training. Cell metabolic and mitochondrial activities were examined using MTT tests [24, 25]. This data is presented as a percentage (control value = 100%). Each test was repeated three times. Calculating the viability percentages involved the following equation:

(1) $$\begin{array}{c} \text{The percentage of cell viability}=\frac{\text{Samples }\left(\text{OD}\right)}{\text{Control }\left(\text{OD}\right)\;}\times 100 \end{array}$$

### 2.7. Antimicrobial Activity of the Propolis Extracts

#### 2.7.1. Bacterial Strain and Inoculum Preparation for Evaluation of MIC and MBC

*Streptococcus mitis*, *S. mutans*, *S. salivarius*, *L. acidophilus*, *S. aureus*, *E. coli*, and *C. albicans* were the bacteria and fungus strains employed in this investigation. In BHI medium (37°C, 5% CO_2_), bacteria are reactivated after 48 hours. A loop of BHI Broth medium (25 mL) was then added to the bacteria (Merck, Darmstadt, Germany). Incubation for 24 hours at 37°C yielded the concentration of cells. In a spectrophotometer (at 625 nm), 1.0108 CFU/mL was measured (absorbance of 0.18) in a spectrophotometer [26]. A sterile YPD broth was used to prepare the suspension of albicans from the stock culture of albicans. In the MIC test, 1.0 × 10^5^ CFU/mL were used [3].

#### 2.7.2. Determination of MIC, MBC, and MFC Tests

A 96-well microtiter plate was injected with 100 mL of BHI broth or YPD broth to determine the MIC. EEP (100 L) was then injected into the wells' first column. A concentration of 15 mg/ml was used. As well content (100 L) was moved from the highest to the lowest concentration [26], the EEP was gradually diluted (1 : 1 v/v) from 15 to 0.007 mg/mL [26]. After the previous column was discarded, 100 liters were added to the new column. A total of 100 L of bacteria and fungi (1.05 105 CFU/mL) were injected in the last step. In these studies, there were three control groups: growth control (only microbiological content) (no antimicrobials), antimicrobial control (CHX 0.2 percent), and sterility control (only sterile culture medium). A temperature of 37°C with 5% CO_2_ was used for incubation of the microplates for 24 hours [3].

#### 2.7.3. Disk Agar Diffusion Test (DAD)

Many strains of bacteria were cultured in BHI and YPD agar and then suspended in NaCl solution. Using McFarland 0.5, they were corrected to spectrophotometric measurement using a spectrophotometer. Propolis suspensions (400 mL) were combined with BHI and YPD agar (40 mL, 45°C). On top of the BHI agar, a layer was added. Inoculations were then made using sterile swabs on plates. YPD agar (3 108/mL concentration) was used to streak strains on BHI agar and YPD agar. For each experiment, 0.08 mL of 2× MBC Propolis, 0.2 percent CHX, and 0.2 percent CHX (positive control) were applied to EEP plates. A 48-hour incubation was carried out at 37°C. An analysis of the inhibition zones was performed [13, 27].

#### 2.7.4. Biofilm Formation and Degradation Evaluation

Biofilm formation was studied using crystal violet staining. Agar plates were cultivated with 1% sucrose and sterilized BHI and YPD agars. Two microplates of each EEP were grown under anaerobic conditions (37°C, 5% CO_2_). In order to remove nonadherent bacteria, we rinsed the microplates with PBS three times after the broths were removed. After forty-five minutes, the microplates were dried at 60°C. After the crystal violet solution was added (100 L, 1% v/v), the reaction was completed. A 15-minute incubation period followed. The microplates were then washed with PBS. 125 *μ*L of ethanol (95 percent) was poured into each well to test the production of biofilms. The optical density of wells was measured at 590 nm using a microplate reader for comparison with a control biofilm (without EEP) [28]. EEP percentage inhibition was calculated for the various concentrations of propolis samples using the following formula: We calculated the mean absorbances of the propolis samples, and the EEP percentage inhibition was calculated for each concentration using the following formula:

(2) $$\begin{array}{c} \text{The biofilm formation rate}=\frac{\text{Samples }\left(\text{OD}\right)}{\text{Control }\left(\text{OD}\right)\;}\times 100 \end{array}$$

(3) $$\begin{array}{c} \text{The biofilm reduction rate}=100-\left(\frac{\text{Samples }\left(\text{OD}\right)}{\text{Control }\left(\text{OD}\right)}\right)\times 100 \end{array}$$

where OD _treatment_ with samples and OD _control_ without samples (570 nm).

### 2.8. Statistical Analysis

An ANOVA of one-way and Tukey post hoc tests were used to compare means between groups. The statistical analysis was carried out using SPSS statistics model 20.

## 3. Results

### 3.1. Determination of Flavonoids and Phenolic Compounds

The obtained results of the flavonoid and phenolic analysis are presented in Table 1. The range of phenolic compounds was from 5575 to 35500 mg/kg. Propolis from Fereydunshahr had the highest phenolic compounds, and propolis from Kermanshah had the lowest phenolic compounds. The range of flavonoids compounds was from 2285 to 63309 mg/kg. Propolis from Khalkhal had the highest flavonoids compounds, and also, propolis from Kermanshah had the lowest flavonoids compounds.

### 3.2. GC/MS Analysis of EEP

**Figure 2:**
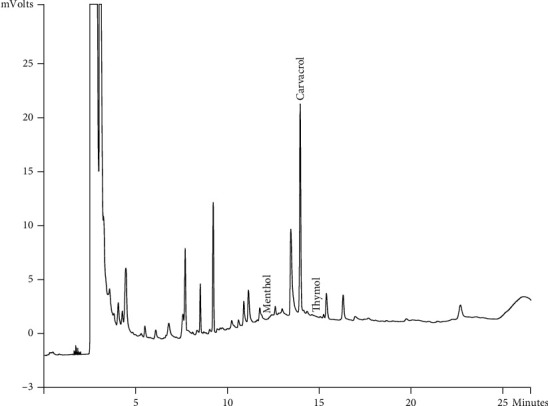
Gas chromatogram of Kermanshah EEPs (GC/MS profile) showing thymol, carvacrol, and menthol as the significant constituents.

**Figure 3:**
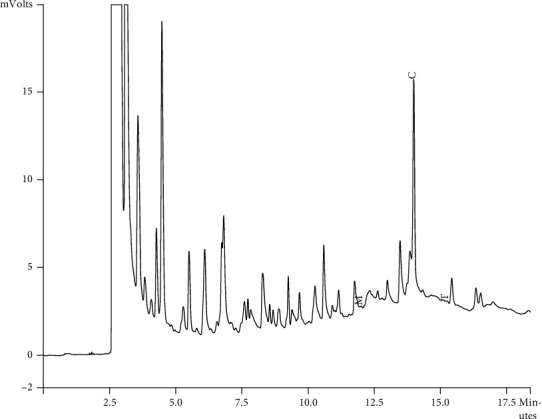
Gas chromatogram of Fasa EEPs (GC/MS profile) showing thymol, carvacrol, and menthol as the significant constituents.

**Figure 4:**
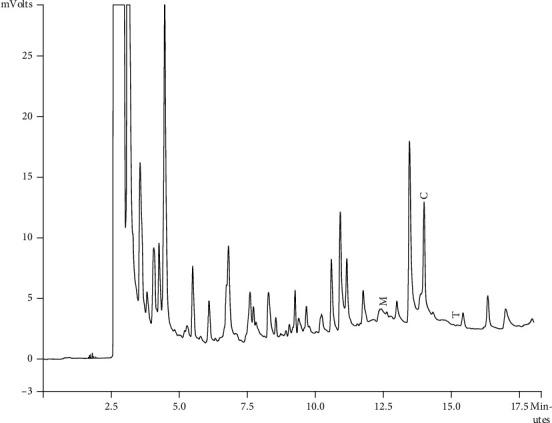
Gas chromatogram of Tabriz EEPs (GC/MS profile) showing thymol, carvacrol, and menthol as the significant constituents.

**Figure 5:**
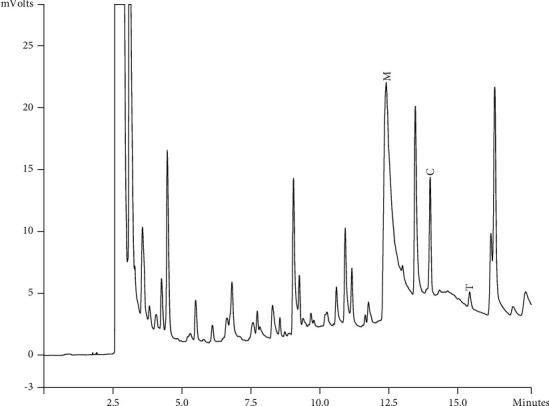
Gas chromatogram of Neor EEPs (GC/MS profile) showing thymol, carvacrol, and menthol as the significant constituents.

**Figure 6:**
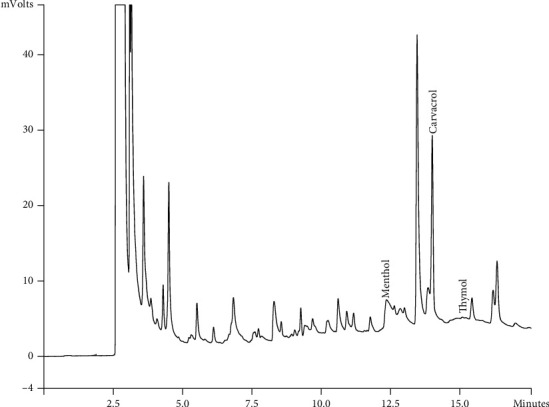
Gas chromatogram of Sarab EEPs (GC/MS profile) showing thymol, carvacrol, and menthol as the significant constituents.

**Figure 7:**
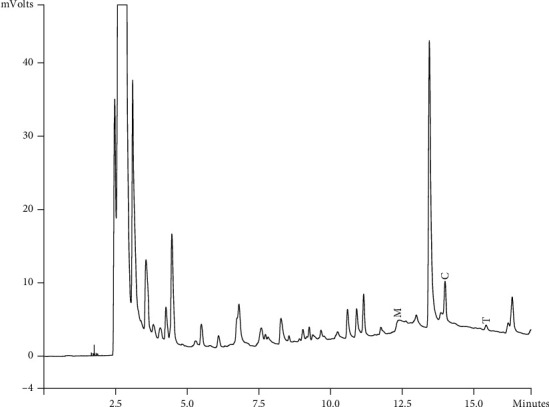
Gas chromatogram of Gilan EEPs (GC/MS profile) showing thymol, carvacrol, and menthol as the significant constituents.

**Figure 8:**
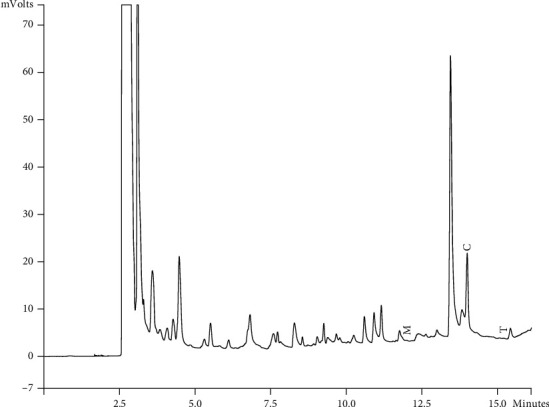
Gas chromatogram of Khalkhal EEPs (GC/MS profile) showing thymol, carvacrol, and menthol as the significant constituents.

**Figure 9:**
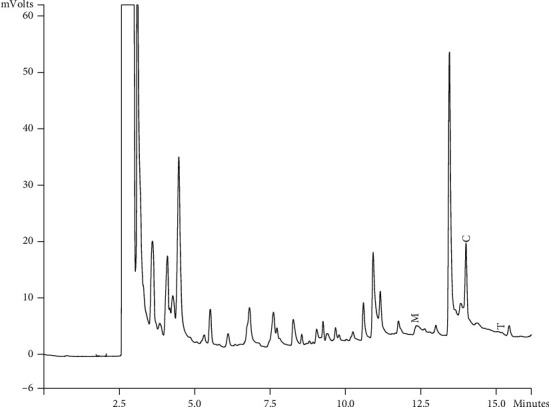
Gas chromatogram of Kurdistan EEPs (GC/MS profile) showing thymol, carvacrol, and menthol as the significant constituents.

**Figure 10:**
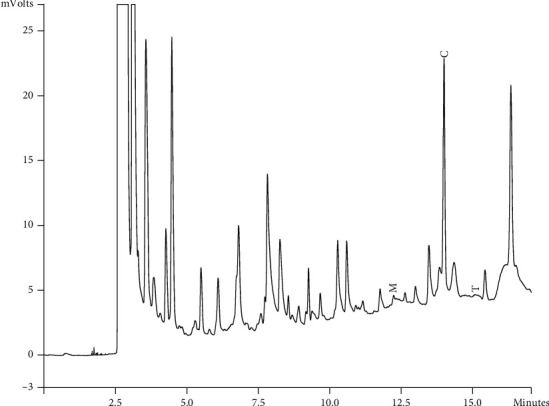
Gas chromatogram of Fereydun Shahr EEPs (GC/MS profile) showing thymol, carvacrol, and menthol as the significant constituents.

Components of different EEPs were recognized including menthol, thymol, carvedilol. The chemical composition of nine extracts was analyzed by GC/MS technique. Figures 2–9 show that the amount of carvacrol was more than thymol and menthol in Kermanshah, Fasa, Tabriz, Sarab, Gilan, Khalkhal, Kurdistan, and Fereydun Shahr EEPs that had the highest amount of carvacrol. In addition, the amount of carvacrol in Kermanshah, Fasa, Sarab, and Fereydun Shahr EEPs was more than Tabriz and Neor EEPs, and also, carvacrol amount in Tabriz and Neor EEPs was more than Gilan, Khalkhal, and Kurdistan EEPs that had the lowest amount of the carvacrol among the samples.  Figure 10 shows that the amount of menthol was more than the carvacrol and thymol in Neor EEPs. Figures 5, 6, 8, and 10 show that the amount of menthol in Neor EEPs was more than Sarab, Gilan, and Kurdistan EEPs. In addition, menthol amount of Sarab, Gilan, and Kurdistan EEPs was more than Kermanshah, Fasa, Tabriz, Khalkhal, and Neor EEPs. The Neor EEPs had the highest amount of menthol among samples. The amount of thymol was more in Kermanshah, Fasa, Tabriz, Neor, Sarab, and Fereydun Shahr EEPs compared to Gilan, Khalkhal, and Kurdistan EEPs (Figure 2; GC of Kermanshah EEPs, Figure 3; GC of Fasa EEPs, Figure 4; GC of Tabriz EEPs, Figure 5; GC of Neor EEPs, Figure 6; GC of Sarab EEPs, Figure 7; GC of Gilan EEPs, Figure 8; GC of Khalkhal EEPs, Figure 9; GC of Kurdistan EEPs, Figure 10; GC of Fereydun Shahr EEPs).

### 3.3. Cell Viability Evaluation

**Figure 11:**
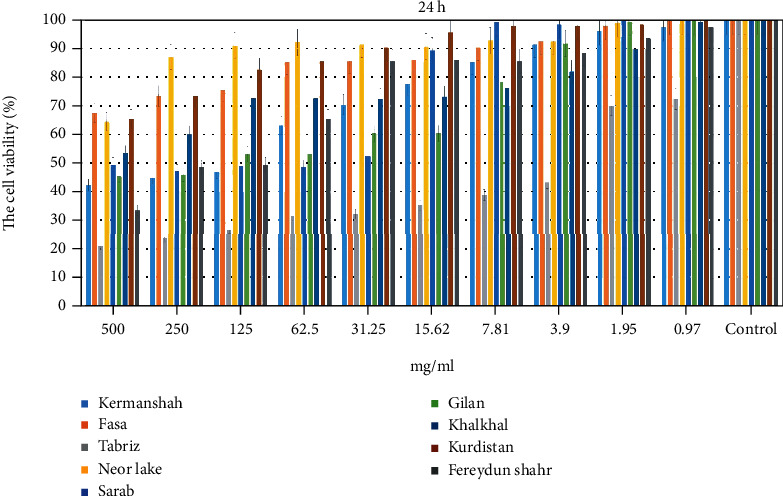
The percentage of cell viability on fibroblast cell lines by MTT assay (24 h). Results are shown as mean ± SD (*n* = 3). 1, Kermanshah; 2, Fasa; 3, Tabriz; 4, Neor Lake; 5, Sarab; 6, Gilan; 7, Khalkhal; 8, Kurdistan; 9, Fereydun Shahr.

**Figure 12:**
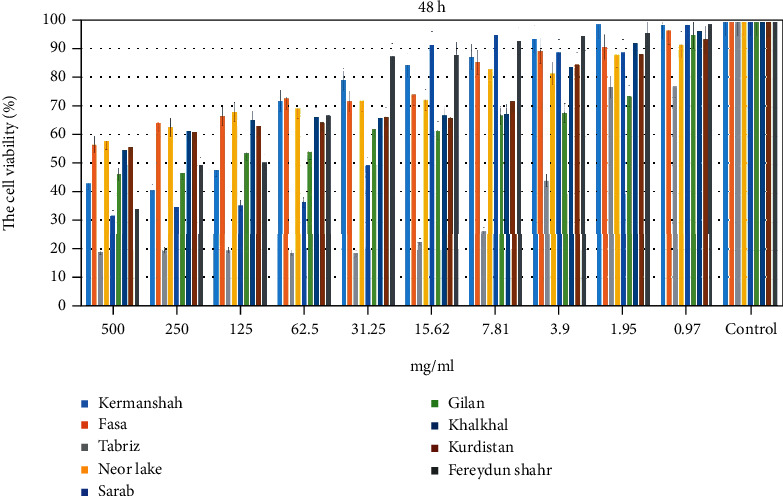
The percentage of cell viability on fibroblast cell lines by MTT assay (48 h). Results are shown as mean ± SD (*n* = 3). 1, Kermanshah; 2, Fasa; 3, Tabriz; 4, Neor Lake; 5, Sarab; 6, Gilan; 7, Khalkhal; 8, Kurdistan; 9, Fereydun Shahr.

Cultured cells were incubated with different extract concentrations (0.97 to 500 mg/mL). Cell viability was determined by the MTT assay. In a dose- and time-dependent manner, extracts significantly reduced the number of viable cells. Following treatment with the samples for incubation durations of 24 and 48 hours, optical density of viable cells was used to calculate the viability percentages for both cell lines and the control group. According to MTT data, the viability of Fasa, Neor Lake, Khalkhal, and Kurdistan propolis was greater than 50% with 500 mg/mL over 24 and 48 hours. Cell viability was also enhanced when all concentrations were reduced. The results are shown in Figures 11 and 12.

### 3.4. Antimicrobial Analysis

#### 3.4.1. MIC

MIC values were calculated using the broth microdilution technique. There was a range in MIC values (mg mL^−1^) (Table 2) for *S. salivarius* (0.003 to 0.048), *S. mutans* (0.003 to 0.029), *S. mitis* (0.007 to 0.058), *L. acidophilus* (0.007 to 0.117), *C. albicans* (0.014 to 0.234), *E. coli* (0.007 to 0.058), and *S. aureus* (0.007 to 0.058) (Table 2). There were statistically difference between groups 2, 4, 5, 6, 7, and 9 with 1, 3, 8 (*P*-value <0.001). There were statistically difference between groups 2, 4, 5, 6, 7, and 9 (*P*-value <0.05). There were statistically difference between groups 1, 3, and 8 (*P*-value <0.05).

#### 3.4.2. MBC and MFC

The range of MBC and MFC values in mg mL^−1^ for *S. mutans* was (0.007 to 0.058), *S. salivarius* (0.007 to 0.117), *S. mitis* (0.007 to 0.117), *L. acidophilus* (0.014 to 0.234), *C. albicans* (0.029 to 0.468), *E. coli* (0.014 to 0.234), and *S. aureus* (0.007 to 0.117 (Table 3). There were statistically difference between groups 2, 4, 5, 6, 7, and 9 with 1, 3, and 8 (*P*-value <0.001). There were statistically difference between groups 2, 4, 5, 6, 7, and 9 (*P*-value <0.05). There were statistically difference between groups 1, 3, and 8 (*P*-value<0.05).

#### 3.4.3. Disk Agar Diffusion Analysis

The results were affected by the strains and EEP samples. As a result, propolis samples inhibited bacterial growth in various zones for *S. mutans* (9.5 to 16), *S. salivarius* (11 to 16), *S. mitis* (7.5 to 17), *L. acidophilus* (9 to 15), *C. albicans* (11 to 13.5), *E. coli* (9 to 15.5), and *S. aureus* (8 to 16) (Table 4). There were statistically difference between groups 2, 4, 5, 6, 7, and 9 with 1, 3, and 8 (*P*-value <0.001). There were statistically difference between groups 2, 4, 5, 6, 7, and 9 (*P*-value<0.05). There was a statistical difference between groups 1, 3, and 8 (*P*-value <0.05).

#### 3.4.4. The Results of Biofilm Formation

To determine whether samples are effective in preventing biofilm development, microdilution was used.  Figure 4 illustrates the percentage of samples that developed biofilm. These percentages are based on comparing the OD of each well with that of the control group (at 570-nm wavelength) in order to assess biofilm formation in the tested microorganisms (Table 5). Propolis sampled from different areas had different antibacterial and antifungal properties. Khalkhal propolis had the highest antibacterial and antifungal properties. On the other hand, Kurdistan, Sarab, and Gilan propolis were ranked after Khalkhal propolis. Tabriz and Neor propolis had fewer antibacterial and antifungal properties than Kurdistan, Sarab, and Gilan Propolis. Kermanshah, Fasa, and Fereydunshahr propolis had the lowest antibacterial and antifungal properties.

#### 3.4.5. The Results of Biofilm Degradation

Biofilms were also investigated by using similar methods. In this case, the biofilm reduction rate was calculated as a percentage (Table 6). Khalkhal propolis had the highest antibacterial and antifungal properties. On the other hand, Kurdistan, Sarab, and Gilan propolis were ranked after Khalkhal propolis. Tabriz, Kermanshah, and Neor propolis had less antibacterial and antifungal properties than Kurdistan, Sarab, and Gilan Propolis. Fasa and Fereydunshahr propolis had the lowest antibacterial and antifungal properties.

## 4. Discussion

Dental caries can be prevented in part by reducing consumption of fermentable carbohydrates, by using fluoride mouthwash, by keeping teeth clean, and by a number of other methods. Caries control coadjutants can also be derived from natural sources. The herbal extract can replace synthetic antimicrobials. Caries is caused by an abundance of bacteria. *S. mutans* is not the only factor related to the onset of caries. In many cases, antibacterial compounds are tested on the biofilm of *S. mutans* [26]. Critical components of natural materials with antimicrobial activities are phenolic compounds. Phenolic compounds inhibit the enzyme glycosyltransferase [29].

Critical components of natural materials with antimicrobial activities are phenolic compounds. Phenolic compounds inhibit the enzyme glycosyltransferase [29]. *S. mutans* uses the enzyme glycosyltransferase to adhere to the tooth surface. The phenolic component artepillin C in propolis is effective against MRSA infections. The extract of propolis kaempferide is used to treat infections caused by *S. mutans*. Quercetin is a flavonoid component of propolis that binds to the DNA gyrase of *E. coli* to delay bacterial activity. Propolis can affect bacterial proteins and cause fractional bacterial lysis. *S. mutans* uses the glycosyltransferase enzyme to stick to the tooth surface. Artepillin C is one of the numerous phenolic components of propolis that showed antibacterial activity against MRSA. Kaempferide is an extract of propolis and is used to treat *S. aureus* skin infections. Also, Kaempferide was highly effective against *E. faecalis*, *S. saprophyticus*, and *L. monocytogenes* [29]. Quercetin is a flavonoid component of propolis that binds to the DNA gyrase of *E. coli* to delay bacterial activity. Proteins in bacteria are altered by propolis, causing partial bacterial lysis. Antibacterial properties were also found for pinocembrin and apigenin in propolis. A variety of microorganisms are resistant to cinnamic acid, which is found in propolis. In addition to damaging bacterial cell membranes, cinnamic acid interferes with ATPase activity, biofilm formation, and bacteria division [29].

In this study, the range of phenolic compounds was from 5.5 to 35.5 mg/g. Propolis from Fereydunshahr has the highest phenolic compounds, and propolis from Kermanshah has the lowest phenolic compounds [26]. The phenolic content of propolis in Osés et al. study was reported from 65.49 to 228.40 (mg GA/g). According to studies, there were different ranges for phenolic contents of propolis extracts depending on solvent and standard used. Using methanol as solvent and gallic acid as standard, total phenolic contents of Portuguese and Brazilian propolis extracts ranged from 29.5 to 137 (mg/g). For propolis from China, Spain, and Poland, by ethanol as solvent and gallic acid as standard, more quantities of phenolic contents were gained 150–340 (mg/g) [30]. The range of flavonoids compounds was from 2.2 to 63.3 mg/g. Propolis from Khalkhal has the highest flavonoids compounds and propolis from Kermanshah has the lowest flavonoids compounds.

Flavonoids compounds were from 18.48 to 83.76 mg (Q/g) in Osés et al.'s study [30]. Our results were similar to other studies from different geographical areas, with results of 13 to 62 (mg Q/g) flavonoids. Similar results were found for Ethiopian propolis extracts from Ethiopia (from 14.76 to 68.88 (mg C/g)), and lower results were found for propolis extracts from Thailand, with an average of 3.40 (mg C/g) [30]. The MIC values were ranged (mg mL^−1^) as follows: *S. salivarius* and *S. mutans* (0.003 to 0.029 and 0.003 to 0.048), *S. mitis* (0.007 to 0.058), *L. acidophilus* (0.007 to 0.117), *C. albicans* (0.014 to 0.234), *E. coli* (0.007 to 0.058), and *S. aureus* (0.007 to 0.058). The MBC and MFC values in mg mL^−1^ were range, respectively: for *S. mutans* (0.007 to 0.058), *S. salivarius* (0.007 to 0.117), *S. mitis* (0.007 to 0.117), *L. acidophilus* (0.014 to 0.234), *C. albicans* (0.029 to 0.468), *E. coli* (0.014 to 0.234), and *S. aureus* (0.007 to 0.117). The values found in this study are lower than those of previous studies [3, 31, 32]. And they are higher than some other studies [1, 5]. The chemical composition of extracts is variable, based on their harvest place, the season of harvest, and the type which cause its various biological properties, for instance, anti-inflammatory, antimicrobial, and antioxidant effects. Thus, these results explain the more study of propolis [26].

Surak et al. (2020) studied the cytotoxic properties of some propolis samples that were investigated by MTT assay on MCF7 (human breast adenocarcinoma), MDA-MB-231 (triple-negative human breast adenocarcinoma), HepG2 (human hepatocellular carcinoma), HeLa (human cervical adenocarcinoma), McCoy (normal mouse fibroblasts) cells, and HRT-18 (human colorectal adenocarcinoma). Propolis was effective against tumor cell lines. They concluded that propolis is a substance with antineoplastic properties [33]. Mohamed et al. (2020) studied the cytotoxic properties of some propolis samples that were investigated by MTT assay on MCF7 and MCF 10A. Propolis was effective against tumor cell lines and inhibited the proliferation of the MCF7 cells [34].

This study, MTT analysis, showed that Fasa, Neor Lake, Khalkhal, and Kurdistan propolis had the highest cell viability with 500 mg/mL during 24 and 48 h. In addition, the cell viability was increased by decreasing the concentration of all groups. In this study, the range of zones of microbial growth inhibition by propolis samples for *S. mutans* was 9.5 to 16; *S. salivarius*, 11 to 16; *S. mitis*, 7.5 to 17; *L. acidophilus*, 9 to 15; *C. albicans*, 11 to 13.5; *E. coli*, 9 to 15.5; and *S. aureus*, 8 to 16. In this study, inhibition zones were higher than in previous studies [13, 35, 36]. We studied the effect of extracts on the degradation and formation of microbial biofilm. Propolis extract from Khalkhal had the highest effect on the formation and degradation, and propolis extract from Fasa had the lowest effect on the degradation and formation of biofilm. In our study, propolis from large areas of Iran was used. The selected areas were located at a considerable distance to study different regions of Iran. Almost all areas where bees were kept and had sufficient vegetation to grow bees and produce bee products were selected. We studied essential microorganisms in oral diseases and other important bacteria.

## 5. Conclusion

Several antimicrobial studies have found that propolis extracts are effective plaque inhibitors and may be used as a mouthwash. By inhibiting plaque development and by reducing biofilm formation, plaques and biofilms were decreased. In order to overcome the disadvantages of the gold chlorhexidine standard, more long-term clinical trials are necessary to incorporate standardization and certification of mouthwash.

## Figures and Tables

**Table 1:** Flavonoid and phenolic contents of the EEPs.

Propolis	Phenolic compounds (mg/kg)	Flavonoids (mg/kg))
Kermanshah	5575	2285
Fasa	35400	10096
Tabriz	14050	35962
Neor lake	19300	22203
Sarab	19500	22705
Gilan	15250	30471
Khalkhal	12000	63309
Kurdistan	16950	33618
Fereydun Shahr	35500	8192

**Table 2:** MIC in mg mL^−1^ of EEP obtained using the broth microdilution method.

Samples	1	2	3	4	5	6	7	8	9	CHX
*S. mutans*	0.003	0.029	0.007	0.029	0.029	0.029	0.029	0.003	0.007	0.0000305
*S. salivarius*	0.003	0.029	0.007	0.029	0.029	0.048	0.048	0.003	0.014	0.0000305
*S. mitis*	0.007	0.029	0.007	0.058	0.058	0.058	0.058	0.014	0.014	0.0000305
*L. acidophilus*	0.007	0.058	0.014	0.058	0.058	0.117	0.058	0.007	0.029	0.0000152
*C. albicans*	0.014	0.117	0.014	0.058	0.234	0.234	0.058	0.029	0.058	0.0000152
*E. coli*	0.007	0.029	0.014	0.058	0.058	0.058	0.058	0.014	0.029	0.0000305
*S. aureus*	0.007	0.029	0.007	0.058	0.048	0.058	0.058	0.014	0.029	0.0000152

∗Kermanshah (sample 1), Fasa (sample 2), Tabriz (sample 3), Neor Lake (sample 4), Sarab (sample 5), Gilan (sample 6), Khalkhal (sample 7), Kurdistan (sample 8), and Fereydun Shahr (sample 9). There were statistically difference between groups 2, 4, 5, 6, 7, and 9 (*P*-value < 0.05). There was a statistical difference between groups 1, 3, and 8 (*P*-value < 0.05). There were statistically difference between groups 2, 4, 5, 6, 7, and 9 with 1, 3, and 8 (*P*-value<0.001).

**Table 3:** MBC and MFC in mg mL^−1^ of EEP obtained using the broth microdilution method.

Samples	1	2	3	4	5	6	7	8	9	CHX
*S. mutans*	0.007	0.058	0.014	0.029	0.058	0.058	0.058	0.007	0.014	0.000244
*S. salivarius*	0.007	0.058	0.014	0.058	0.058	0.117	0.117	0.007	0.029	0.000244
*S. mitis*	0.014	0.058	0.007	0.117	0.029	0.117	0.117	0.014	0.029	0.000244
*L. acidophilus*	0.014	0.117	0.029	0.117	0.117	0.234	0.117	0.014	0.058	0.000122
*C. albicans*	0.058	0.234	0.029	0.234	0.468	0.468	0.117	0.058	0.117	0.000976
*E. coli*	0.014	0.117	0.014	0.117	0.117	0.234	0.117	0.014	0.058	0.000122
*S. aureus*	0.014	0.058	0.007	0.058	0.029	0.117	0.117	0.014	0.029	0.000122

**Table 4:** Mean area of microbial growth inhibition zones in mm (*n* = 3) provided by the EEP samples.

Samples	2 × MBC concentrations of each Propolis									CHX 0.2%
	1	2	3	4	5	6	7	8	9	
*S. mutans*	9.5	16	14	15	15	15.5	16	10	14.5	20
*S. salivarius*	11	14	12	14	14	15	16	11	13	20
*S. mitis*	12.5	15	7.5	16.5	14	15.5	17	12	14.5	19.5
*L. acidophilus*	10.5	13.5	11	13.5	14	15	14	9	12	21
*C. albicans*	10.5	12	9.5	12.5	13.5	13	11.5	12	11	19
*E. coli*	10.5	14	11	13.5	14	15.5	14	9	11.5	20
*S. aureus*	12	15	8	16	14	15.5	16	12	14	20

∗Kermanshah (sample 1), Fasa (sample 2), Tabriz (sample 3), Neor Lake (sample 4), Sarab (sample 5), Gilan (sample 6), Khalkhal (sample 7), Kurdistan (sample 8), and Fereydun Shahr (sample 9). There were statistically difference between groups 2, 4, 5, 6, 7, and 9 (*P*-value < 0.05). There was a statistical difference between groups 1, 3, and 8 (*P*-value < 0.05). There were statistically difference between groups 2, 4, 5, 6, 7, and 9 with 1, 3, and 8 (*P*-value<0.001).

**Table 5:** The percentage of microbial biofilm formation.

Samples	2 × MBC/MFC concentrations of each Propolis %									CHX 0.2%
	1	2	3	4	5	6	7	8	9	
*S. mutans*	30	4	23.5	24	37.5	38	41	29	6.5	86
*S. salivarius*	24.5%	7	24	22.5	44	32	42.5	39	8.5	84.5
*S. mitis*	46	16	27	31	44	32	51	42.5	9.5	85
*L. acidophilus*	33	28.5	31	31.5	39	38.5	44	40	28	83
*C. albicans*	8	2.5	8	1	8.5	11	13	2.5	2.5	82.5
*E. coli*	30	28	31	31	36	38	44	42	26	84
*S. aureus*	45	16	27	31	42	33	51	42	9.5	84

Kermanshah (sample 1), Fasa (sample 2), Tabriz (sample 3), Neor Lake (sample 4), Sarab (sample 5), Gilan (sample 6), Khalkhal (sample 7), Kurdistan (sample 8), and Fereydun Shahr (sample 9). There were statistically difference between groups 2, 4, 5, 6, 7, and 9 (*P*-value < 0.05). There was a statistical difference between groups 1, 3, and 8 (*P*-value<0.05). There were statistically difference between groups 2, 4, 5, 6, 7, and 9 with 1, 3, and 8 (*P*-value < 0.001).

**Table 6:** The percentage of microbial biofilm degradation.

Samples	2 × MBC/MFC concentrations of each Propolis									CHX 0.2
	1	2	3	4	5	6	7	8	9	
*S. mutans*	27%	5	21	21	23	27.5	30.5	23	3.5	75%
*S. salivarius*	16.5	4.5	17	16.5	27.5	20	28	26.5	4.5	79
*S. mitis*	17	5	13.5	13.5	22.5	17	65.5	23	3.5	75.5
*L. acidophilus*	13.5	7.5	17	13.5	21	18.5	22.5	25	4.5	73
*C. albicans*	12	2.5	5.5	4.5	9.5	15	15	2.5	4	74
*E. coli*	13	7.5	16	14	22	16	23	24	4	73
*S. aureus*	16	5.5	13	14	22	16	60	21	4	74

∗Kermanshah (sample 1), Fasa (sample 2), Tabriz (sample 3), Neor Lake (sample 4), Sarab (sample 5), Gilan (sample 6), Khalkhal (sample 7), Kurdistan (sample 8), and Fereydun Shahr (sample 9). There were statistically difference between groups 2, 4, 5, 6, 7, and 9 (*P*-value < 0.05). There was a statistical difference between groups 1, 3, and 8 (*P*-value < 0.05). There were statistically difference between groups 2, 4, 5, 6, 7, and 9 with 1, 3, and 8 (*P*-value <Kermanshah (sample 1), Fasa (sample 2), Tabriz (sample 3), Neor Lake (sample 4), Sarab (sample 5), Gilan (sample 6), Khalkhal (sample 7), Kurdistan (sample 8), and Fereydun Shahr (sample 9). There were statistically difference between groups 2, 4, 5, 6, 7, and 9 (*P*-value < 0.05). There was a statistical difference between groups 1, 3, and 8 (*P*-value < 0.05). There were statistically difference between groups 2, 4, 5, 6, 7, and 9 with 1, 3, and 8 (*P*-value < 0.001). 0.001).

## Data Availability

All the data generated or analyzed during this study are included in this article, and also, the datasets analyzed to support the findings of this study are available from the corresponding author upon request.
